# Task demands modulate distal limb handedness: A comparative analysis of prehensile synergies of the dominant and non-dominant hand

**DOI:** 10.1038/s41598-024-75001-3

**Published:** 2024-10-26

**Authors:** Prajwal Shenoy, Varadhan S. K. M.

**Affiliations:** 1 Department of Mechatronics, Manipal Institute of Technology, Manipal Academy of Higher Education (MAHE), Manipal, Karnataka 576104 India; 2 Department of Applied Mechanics and Biomedical Engineering, Indian Institute of Technology Madras, Chennai, Tamil Nadu 600036 India

**Keywords:** Biomedical engineering, Motor control

## Abstract

The dynamic dominance hypothesis of handedness suggests a distinct control strategy for the dominant and the non-dominant limb. The hypothesis demonstrated that the dominant proximal limb is tuned for optimal trajectory control while the non-dominant limb is tuned for a stable grasp. Whether the hypothesis can be extended to distal segments like fingers, especially during a five-fingered grasp, has been studied little. To examine this, an attempt was made to compare the prehensile synergies and force magnitudes of the dominant (DOM) and non-dominant hands (NDOM) during a 5-fingered prehension task. Participants traced a trapezoidal and inverse trapezoidal path with their thumbs on a sliding platform while holding a handle in static equilibrium. The DOM hand performed better only in the inverse trapezoid condition, exhibiting a reduced grip force and increased synergy index aligning with the dynamic dominance hypothesis. No differences were observed for the trapezoid condition, likely due to reduced task demands. The study also explored changes in anticipatory synergy adjustments between the DOM and NDOM hands, but the differences were non-significant. Overall, the DOM hand demonstrated better force coordination than the NDOM hand in challenging conditions. Applications of the study in the objective assessment of handedness were proposed.

**Supplementary Information:**

The online version contains supplementary material available at 10.1038/s41598-024-75001-3.

## Introduction

The human hand is a dextrous manipulator that allows us to grasp as well as to manipulate a variety of objects. From the rigid grasping of a tool to the delicate handling of an egg, the human hand coordinates the forces of the fingertips for efficient grasping and handling of objects. How the Central Nervous System (CNS) coordinates the forces to maintain the equilibrium of the handheld object has been an active area of research in the last few decades. Researchers have previously designed and performed grasping experiments on custom-made multi-finger handles of different grip configurations to study this. Multi-finger precision involves the participation of all fingers and the thumb to attain the static equilibrium of an object positioned vertically in the air. The physical aspects of these handles are modified in terms of object orientation^1,2^, weight^3,4^, friction^5–7^, and application of external torque^8,9^ to mimic real-life objects and simulate the effects of external perturbation. The measured forces and moments, specifically the grip force (force normal to the sensor surface), load force (force opposite to gravity), and moments due to both the grip force and tangential forces, are then used to obtain synergy index—a metric that indicates the extent of coordination between the fingers. A higher positive synergy value indicates a greater negative covariation among individual finger forces and moments (elemental variables) that minimizes the variability in key variables like total normal force or moments or load forces (performance variables)^10,11^.

An area studied little is the force coordination or synergies between the dominant hand (DOM) and non-dominant (NDOM) hand. Neuroscience research has proposed the dynamic dominance hypothesis for handedness, according to which the dominant and non-dominant hemispheres/limbs are specialized for different features of task performance^12,13^. The DOM limb is associated with optimal trajectory control, whereas the NDOM limb is associated with maintaining the stability of the grasped object^14^. For example, when pouring water from a bottle into a glass, the DOM limb is tuned to ensure the accurate tilting of the bottle, whereas the NDOM limb holds the glass steady. In other words, the NDOM limb is functionally not inferior but is special in its own way, which helps in successfully executing the task. While such a hypothesis has been documented for arm movements, the effect of handedness on finger forces and kinematics has been studied little. An expectation of differences in the extent of asymmetries between the arm and finger movements arises due to the difference in the cortical control of these parts. The distal segments have a greater number of monosynaptic projections from the cortical neurons that innervate the hand muscles than proximal segments^15–17^. The latter is mainly due to activation through spinal interneuronal circuits, but the results could be different for the distal hand segments due to direct cortical control.

Previous studies evaluating the performance of DOM and NDOM hands have been presented in the force domain through pressing tasks^18,19^, finger enslavement analysis^20–23^, and multidigit prehension^24^. On similar lines, comparative analysis has been performed in the kinematic domain^25,26^ and the muscle synergy space^27^. A test for generalizing the dynamic dominance hypothesis to the finger forces has been performed previously using an isometric 4-finger pressing task^18^. The results showed that performance in terms of force amplitudes was the same between the DOM and NDOM hand, but the NDOM hand showed a greater drop in synergy index during force increase. No advantage of the NDOM hand was observed while applying a steady force. However, another study observed an increase in the synergy index of the NDOM hand during steady-state force production tasks^19^. Similarly, another study involving constant force production task observed differences between the DOM and NDOM hands in synergies and timing of anticipatory synergy adjustments^28^. The results of these experiments suggest that the dynamic dominance hypothesis demonstrated for the proximal limbs could be extended to the hand and finger space. While these studies employed finger-pressing tasks, actual tasks in real-life scenarios utilize multidigit prehension involving simultaneous use of the finger and the thumb forces. Along these lines, an experiment involving handling fragile objects using multidigit prehension was performed^24^. An arrangement that collapses upon applying a force greater than a particular threshold was used. Surprisingly, the results showed no significant difference between the DOM and NDOM hands regarding peak acceleration, movement time, grip force, and safety margin while grasping a multi-finger handle. Several other studies, including the analysis of muscle synergies^27^, comparison of kinematic synergies^26^, and study of enslaving effects^20–23^, have not been able to establish any differences in measured DOM and NDOM hand parameters, even though sufficient evidence suggests a different control strategy between these two hands.

This study will assess whether the dynamic dominance hypothesis can be expanded to multi-finger prismatic prehension tasks, given the limited literature and mixed results on DOM and NDOM hand control. To do this, we compare the force amplitude and prehensile synergies of the DOM and NDOM hand during a five-fingered prismatic precision grasp on a handle with an unstable thumb platform. In this setup, five sensors measure the forces and moments of the Index(I), Middle (M), ring (R), and little (L) fingers along the X, Y, and Z axes. The thumb sensor can slide on a slider between the index and little fingers^29,30^. The current study is based on our previous work on the same handle using the DOM hand^30^, where the participants had to trace a trapezoidal and inverse trapezoidal pattern with their thumb while holding the handle vertically and steadily. In other words, the thumb moved the slider towards the index finger (trapezoidal pattern) and the little finger (inverse trapezoidal pattern). Due to a lack of vertical support to the thumb, the ability of the thumb to exert a tangential force is diminished, resulting in an imbalance in the moments^29^. How the finger forces vary to restore the balance was studied. The results showed that when the thumb moved towards the little finger, the increase in the normal force of the little finger to compensate for the lack of thumb tangential force was significantly higher than the increase in the normal force of the index finger when the thumb moved toward the index finger. This was attributed to morphological reasons, which make the operation of the thumb in the vicinity of the little finger challenging, thus increasing task demands as it operates in an extreme range of motion. Thus, tracing the inverse trapezoidal pattern was perceived as challenging, resulting in greater application of normal forces. However, the synergies were not studied.

This study will follow a similar protocol for the DOM and NDOM hands separately. How the fingers of the DOM and the NDOM hands coordinate in restoring equilibrium will be studied by comparing their forces and synergies for trapezoidal and inverse trapezoidal patterns. Additionally, the ability of the DOM and NDOM hands to adapt to destabilizing movements in advance and maintain stability will be studied through the analysis of anticipatory synergy adjustments (ASAs). Considering the task difficulty, we hypothesize that the DOM hand will produce a lesser absolute force and exhibit an increased synergy index compared to the NDOM hand since the NDOM is not trained to make fine and challenging manipulations and must grasp harder to achieve the task. This study does not test the stability component of the NDOM hand of the dynamic dominance hypothesis.

## Materials and methods

### Participants

Fourteen right-handed participants (Eight male and Six female, Age ± S.D = 29 ± 4.03) participated in the study. Written informed consent was obtained from the participants before the commencement of the study. They reported no history of neuromotor disorders or arm or hand injury. Handedness was determined using the Edinburgh handedness inventory score evaluated using the original 20-point questionnaire^31^. Based on the analysis of the questionnaire results, all participants were reported to be right-handed. The experimental procedure was approved by the Institutional Human Ethics Committee (IITM-IHEC) of the Indian Institute of Technology Madras (Approval Number: IEC/2022-03/SKM/01). All the experimental sessions were performed in accordance with the procedures approved by the Institute Human Ethics Committee of the Indian Institute of Technology Madras.

### Experimental setup

**Fig. 1:**
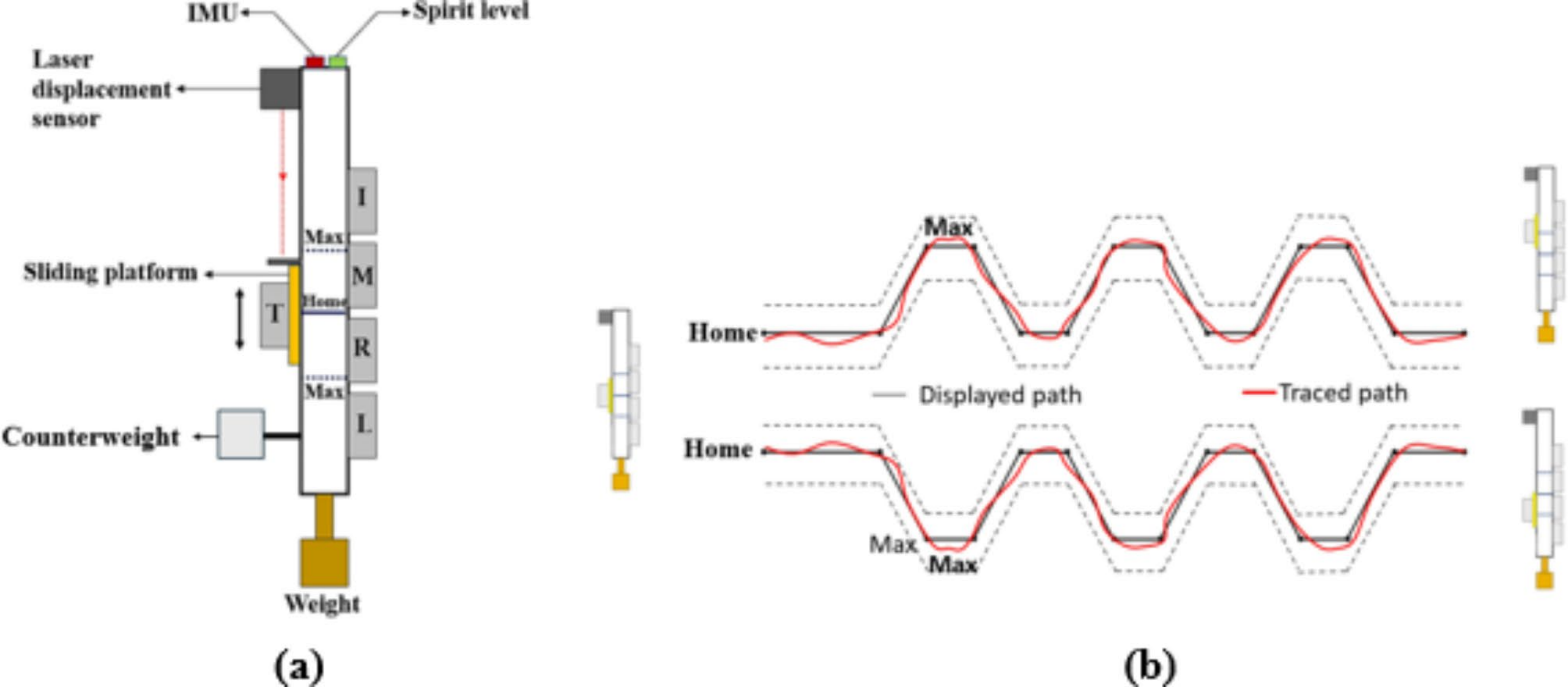
(**a**) Schematic representation of the five-finger grasping handle with force sensors for Thumb (T), Index (I), Middle (M), Ring (R), and Little (L) fingers. The thumb sensor is attached to a sliding platform. A counterweight made of a rectangular aluminium block is attached at the bottom of the handle. A laser displacement sensor measures the displacement of the slider. HOME and MAX are marked, referring to the slider platform’s static positions. During the trapezoid condition, the MAX position at the top acts as a limit; for the inverse trapezoid condition, the MAX position at the bottom acts as a limit. BNO055 IMU is used to measure the tilt of the handle. A spirit level is attached to enable the participant to maintain the vertical equilibrium of the handle. (**b**) Schematic of the tasks to be performed. In the first task, the thumb slider is moved to trace a trapezoidal path that varies from the HOME position to the MAX position (15 mm). In the second task, an inverse trapezoidal pattern is traced between the HOME and MAX positions (15 mm) at the bottom.

A five-fingered precision handle fabricated using aluminium was used for the experiment. The handle is mounted with five force/torque sensors that measure forces and moments about the sensor’s X, Y, and Z axes, respectively. The sensors used in this study are Nano 17, ATI Industrial Automation, NC, USA, with a force resolution of 0.0125 N in the normal and tangential directions. This is shown in Fig. 1a. The thumb sensor was attached to a slider via a baseplate, and the sensors for other fingers were mounted directly to the handle via a baseplate. A laser displacement sensor with a resolution of 5 μm (Baumer, India. Model: OADM 12U6460) was mounted on the top of the handle to track the thumb slider position. A spirit level with a bull’s eye was attached to the handle at the top so the participant could maintain the handle in equilibrium. A BNO055 IMU (Bosch Sensortec) was attached to measure the handle’s orientation. The total weight of the handle was 750 g. A counterweight made of aluminium with a mass of 0.035 kg was attached at the bottom of the handle to shift the center of mass due to an imbalance in the number of sensors on either side of the handle. An additional dimensional description of the experimental apparatus is provided in our previous study^30^. The participants were required to trace a trapezoid and inverse trapezoid pattern with the thumb slider (Fig. 1b). The data from force/torque sensors, IMU, and Baumer displacement sensors were synchronized and collected through a customized LABVIEW program at a sampling frequency of 100 Hz.

### Experimental procedure

The participants had their forearms supinated at 90° and were comfortably seated on a wooden chair. The experiment involved 2 tasks: Tracing a trapezoid and an inverse trapezoid pattern using the thumb slider. To do this, the trapezoid pattern to be traced was displayed on a monitor in front of the participant (see Fig. 1b). Acceptable error margins were displayed using dotted lines above and below the main trapezoid patterns. The error margins were 0.5 cm above and below the main trapezoidal path. In both tasks, the participants were required to translate the thumb slider from the HOME position to the MAX position at 1.5 cm. The laser displacement sensor was used to measure the position of the thumb, which provided feedback to the participants regarding the traced path.

For the first 5 s, the participants held the handle in the air with the thumb slider in the home position using their DOM hand. Following this, they were required to move the slider along an up ramp, hold at the MAX position for 2 s, and slide down along a down ramp in the HOME position for 2 s. Each trial involved tracing 3 such trapezoids, and the entire trial lasted 30 s before returning the handle to its suspended state. The participants repeated each trial 12 times. The same procedure was carried out for the second task involving inverse trapezoids. A 1-min break was provided between each trial and a 10-min break after the first task. The same instructions were repeated for the NDOM hand in a separate session within 2 weeks. The order of hands (DOM or NDOM) and condition (Trapezoid or inverse trapezoid) was balanced across participants. A summary is provided in Table 1.

**Table 1:** Experimental protocol used in the study. The table depicts the tasks, trials, and number of trapezoids used per trial for each DOM and NDOM hand.

Condition number	Hand	Task	Trapezoids per trial	Number of trials	Total number of trapezoids
1.	DOM	Trapezoid	3	12	36
10 min break					
2.	DOM	Inverse Trapezoid	3	12	36
Break up to 2 weeks.					
3.	NDOM	Trapezoid	3	12	36
10 min break					
4.	NDOM	Inverse Trapezoid	3	12	36

### Data analysis

Data obtained from the force/torque and laser displacement sensors was filtered using a Butterworth filter with a cut-off frequency of 15 Hz. The filter used was of second order, low pass, and with a zero phase lag. Force data of the first second of static flat regions of MAX and HOME of both trapezoid and inverted trapezoid paths were analyzed for the DOM and NDOM hands separately. Each DOM and NDOM hand trial consisted of 3 HOME and 3 MAX trials. Hence, each participant’s data included 36 segments with 12 trials, 3 segments per trial for the trapezoid condition, and 36 similar segments for inverse trapezoid conditions.

#### Visualization of thumb displacement and computation of handle orientation

The thumb displacement data for the entire traced path for trapezoid and inverse trapezoid conditions were averaged for each trial and then across all participants. The mean traced path for DOM and NDOM hands with S.E.M was displayed. The analysis was performed to examine the accuracy with which the participants traced the thumb displacement pattern.

The IMU was programmed to produce quaternion output to compute the handle orientation. Before beginning the experiment, the experimenter oriented the handle to zero degrees for 5 s along the X, Y, and Z axes with the help of a bull’s eye. The quaternions were stored and averaged to produce the reference quaternion $\:\left({Q}_{reference}\right)$. During the experiment trials, the measured quaternions ($\:{Q}_{trial})$ were represented relative to the reference quaternion using the quaternion conjugate operation (1).

1 $$\:{Q}_{relative}={{Q}_{reference}}^{conj}\otimes\:{Q}_{trial}$$

The relative quaternions were utilized to obtain the net tilt of the handle. The tilt angles are averaged in the static regions and presented. No orientation feedback was provided to the participants during the trial. The participants initially relied only on the bull’s eye to orient the handle in equilibrium. The main analysis in the study involves the analysis of synergy indices computed from three parameters, namely normal forces exerted by the fingers, tangential forces, and the moments of these forces. The analysis assumes static conditions wherein these forces and moments balance each other. These conditions are only valid when the tilt of the handle is small. For this purpose, the tilt of the handle was monitored, and a given trial was discarded if the tilt exceeded 3$^\circ\:$.

#### Analysis of normal and tangential forces

Normal and tangential forces of the static regions of HOME and MAX were averaged across samples and participants separately for 4 conditions mentioned in Table 1. Average values with standard error of the mean are presented.

#### Analysis of multi-finger synergies

The synergy index is computed for 3 quantities based on the three equations of equilibrium:

- The normal force of the virtual finger should be equal to the normal force of the thumb.
- The sum of the tangential force of the thumb and the virtual finger should equal the weight of the object lifted.
- Total moment of forces produced by the virtual finger must equal the total moment of forces produced by the thumb.

Variance analysis is performed at the VF-TH (Virtual finger-Thumb) level to compute the synergy index. The synergy index $\:\varDelta\:V$ is computed using Eq. (2), as previously discussed in previous literature^32,33^.

2 $$\:\varDelta\:V=\frac{\sum\:Var\:\left(EVs\right)-Var\:\left(PV\right)}{\sum\:Var\:\left(EVs\right)}$$

Here, EVs represent elemental variables that form the right-hand side of Eqs. (3)–(5), and PV represents the performance variables that form the left-hand side of the corresponding equations. In other words, $\:\sum\:Var\:\left(EVs\right)$ represents the variance of the sum of individual forces or moments and $\:Var\:\left(PV\right)$ represents the variance of their corresponding combined output [$\:Var\:\left(\sum\:EVs\right)$].

3 $$\:{F}^{N}={F}_{VF}^{N}+{F}_{TH}^{N}$$

4 $$\:{F}^{T}={F}_{VF}^{T}+{F}_{TH}^{T}$$

5 $$\:{M}_{TOT}={M}_{VF}^{N}+{M}_{TH}^{N}+{M}_{VF}^{T}+{M}_{TH}^{T}$$

Here, Superscripts N and T indicate normal and tangential forces, respectively. Subscripts VF and TH indicate virtual finger and thumb, respectively. M refers to moments computed along corresponding axes for the normal and tangential directions. MTOT refers to the total moment.

The synergy index $\:(\varDelta\:V)$ was computed for each point in time for all participants. A positive value of the synergy index indicates a negative covariation, which indicates a synergy that stabilizes the performance variables. The synergy index was averaged across all participants for visualization. This was done to examine the variation of synergy indices as the participants performed the thumb movements. For statistical comparison, Fisher’s Z transform (6) was performed on the positive values of $\:\varDelta\:V$.

6 $$\:{\varDelta\:V}_{Z}=0.5\:\text{l}\text{n}\left(\frac{1+\varDelta\:V}{1-\varDelta\:V}\right)$$

The synergy index $\:{\varDelta\:V}_{Z}$ was averaged separately across participants in the static regions of HOME and MAX for all 4 conditions mentioned in Table 1. The mean was reported with the standard error of means. $\:{\varDelta\:V}_{Z}\:$was computed for each participant’s DOM and NDOM hands.

The synergy index $\:{\varDelta\:V}_{Z}$ was averaged separately across participants in the static regions of HOME and MAX for all 4 conditions mentioned in Table 1. The mean was reported with the standard error of means. $\:{\varDelta\:V}_{Z}\:$was computed for each participant’s DOM and NDOM hands.

#### Analysis of anticipatory synergy adjustments

Even though a higher value of the synergy index represents better finger-force coordination, a drop in the synergy index is sometimes observed ahead of movement initiation. Such a drop has been attributed to anticipatory synergy adjustments (ASA), which happen in anticipation of destabilizing forces^34,35^. It dictates the ability of the fingers to adapt and coordinate in advance to maintain stability and precision during various tasks. The greater the drop, the better the ability of the fingers to coordinate future perturbations. The ASA was computed using Eq. (7).

7 $$\Delta \Delta Vz=(\Delta Vz){\text{j}} - (\Delta Vz){\text{i}}$$

Here, (Δ*Vz*)j represents the Z-transformed synergy index for one second after the movement initiation of a trapezoid, and (Δ*Vz*)i represents the Z-transformed synergy index for one second before the movement initiation. The index ΔΔ *Vz* was averaged across all participants and reported.

#### Statistics

Statistical tests were performed for the trapezoid and inverse trapezoid conditions in all cases separately using R. For analyzing the absolute normal and tangential forces, two-way ANOVA was performed with factors such as fingers (Levels: Index, Middle, Ring, and Little) and position-hand conditions (DOM-MAX, NDOM-MAX, DOM-HOME, and NDOM-HOME). Two-way ANOVA was performed to analyze synergies on the Z-transformed synergy indices of the tangential forces and total moments. The factors used were position (HOME, MAX) and hand (DOM and NDOM). Since the thumb’s normal force depends on the individual finger’s normal forces, a separate one-way ANOVA was performed on the thumb’s normal force. The factor used was position-hand conditions (DOM-MAX, NDOM-MAX, DOM-HOME, and NDOM-HOME). A sphericity test was performed, and the Huynh–Feldt (H–F) criterion was used wherever applicable to adjust the number of degrees of freedom. A pairwise post hoc Tukey test was performed to determine the significance within factors. Partial eta-squared (η2) was reported as effect size.

## Results

### Visualization of thumb displacement

**Fig. 2:**
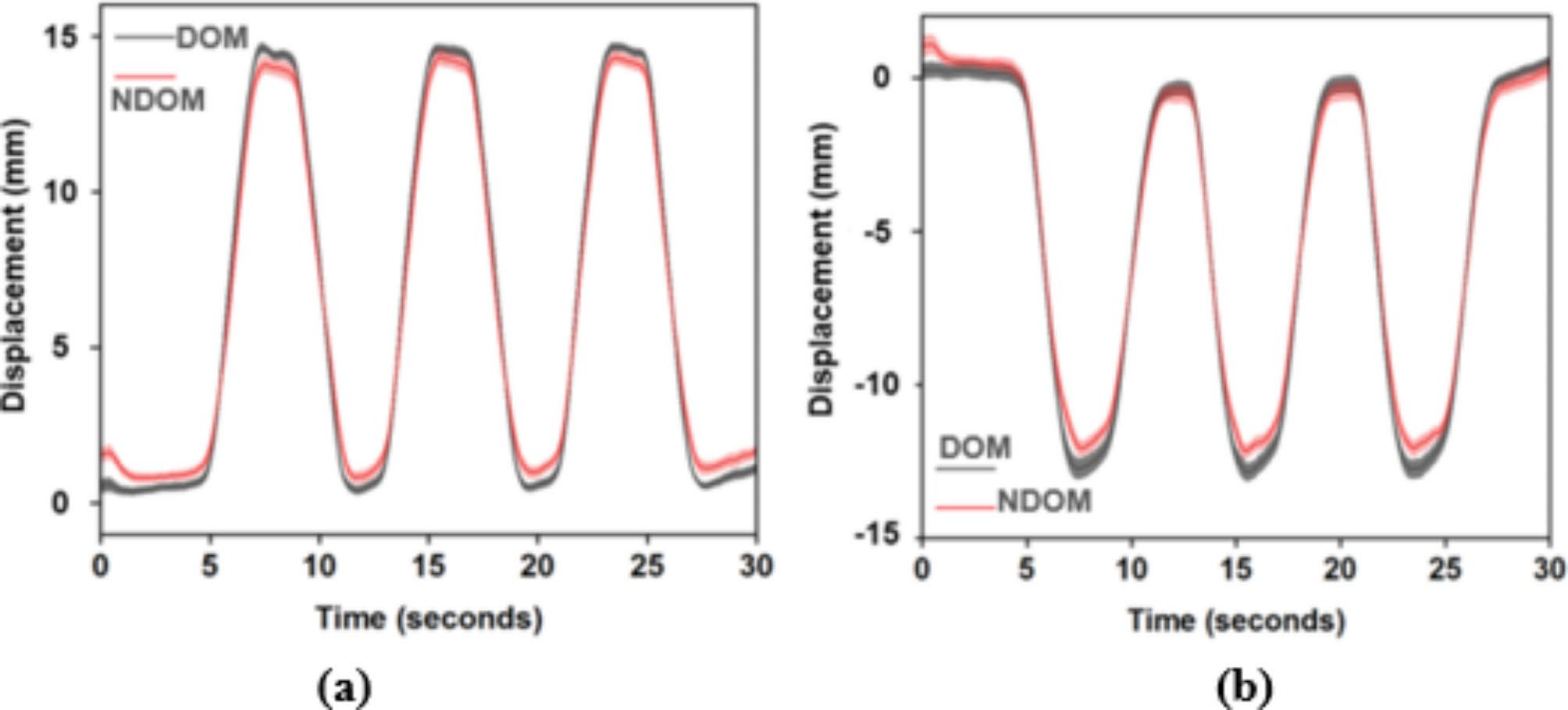
Average displacement of thumb for (**a**) Trapezoid and (**b**) Inverse trapezoid condition. The curves represent the average displacement (averaged across trials and then across participants), while the shaded region indicates S.E.M.

Participants traced the patterns of the trapezoid and inverse trapezoidal paths within the displayed error margin, as shown in Fig. 2a,b for the trapezoid and inverse trapezoid conditions, respectively. The shaded regions indicate S.E.M. Additionally, it was ensured that the participants maintained the handle in static equilibrium. The average tilt (mean ± S.E.M) for DOM-trapezoid, NDOM-trapezoid, DOM-inverse trapezoid and NDOM-inverse trapezoid computed using the IMU is reported in Table 2.

**Table 2:** Net tilt of the handle averaged across participants for different conditions in the static regions of HOME and MAX.

Condition number	Hand	Task	Net Tilt (degrees) (Mean$\:\pm\:{S}.{E}.{M}$)
1.	DOM	Trapezoid	1.36 ± 0.095
2.	DOM	Inverse trapezoid	1.75 ± 0.088
3.	NDOM	Trapezoid	1.97 ± 0.097
4.	NDOM	Inverse trapezoid	2.02 ± 0.095

### Analysis of normal and tangential forces

**Fig. 3:**
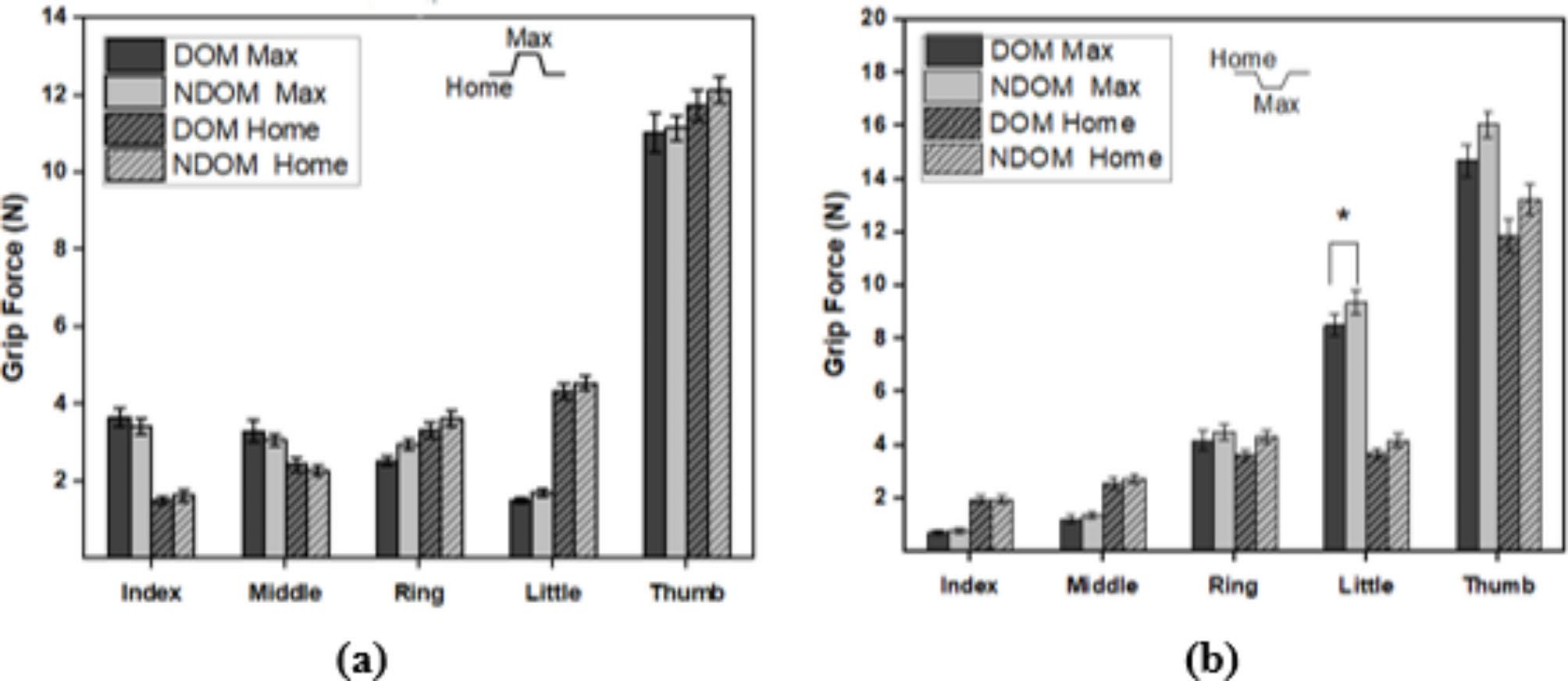
Average values of normal forces for fingers and the thumb for (**a**) Trapezoid and (**b**) Inverse trapezoid condition. Results are presented for the MAX and HOME positions for DOM and NDOM hands. Error bars represent S.E.M.

The normal forces for the trapezoid and inverse trapezoid tasks are shown in Fig. 3a, b. The results showed no difference between the normal forces of the DOM and NDOM hands for the trapezoid task for any fingers or the thumb at both HOME and MAX positions. However, for the inverse trapezoid task, the results showed that the little finger of the NDOM hand exerted greater normal force than the DOM hand at the MAX position. There was a statistically notable effect on the factor fingers for the trapezoid task (F_(3,11)_ = 5.30, *p* < 0.05, *η*^2^ = 0.28) as well as for the inverse trapezoid task (F_(3,11)_ = 232.63, *p* < 0.01, *η*^2^ = 0.947). The ANOVA results also showed a significant effect of factor position-hand conditions for the trapezoid task (F_(3,11)_ = 4.48, *p* < 0.05, *η*^2^ = 0.25) as well as the inverse trapezoid task (F_(3,11)_ = 23.18, *p* < 0.01, *η*2 = 0.638). For the trapezoid task, the interactions of factors fingers and position-hand conditions showed a substantial effect (F_(3,11)_ = 76.73, *p* < 0.05, *η*^2^ = 0.85). Still, a post hoc Tukey test revealed no considerable difference between the hands (DOM and NDOM) in MAX and HOME conditions. Whereas, for the inverse trapezoid condition, a post hoc Tukey test showed that the grip force of the little finger of the NDOM hand was higher (*p* < 0.05) than that of the NDOM hand at the MAX position (DOM_little: mean = 8.5, S.E.M = 0.38 NDOM_little: mean = 9.4, S.E.M = 0.43). A one-way ANOVA performed on the thumb normal forces showed that there was a considerable effect of factor Thumb for the trapezoid task (F_(3,11)_ = 5.409, *p* < 0.05, *η*^2^ = 0.29) and also in the inverse trapezoid task (F_(3,11)_ = 24.35, *p* < 0.01, *η*^2^ = 0.653). Still, a post hoc Tukey test revealed no notable difference between the thumb normal forces of the DOM and NDOM hands in both HOME and MAX conditions.

For the tangential forces, the results showed no difference in both trapezoid and inverse trapezoid tasks at HOME and MAX positions between the DOM and NDOM hands. This is presented in supplementary material (see Supplementary Fig S1a, b). Thus, in the absence of force feedback, the amplitudes of exerted grip and tangential forces were not different in all conditions except for the grip forces of the little fingers in the inverse trapezoid condition.

### Analysis of multi-finger synergies

The z-transformed synergy index Δ*Vz* computed for the normal forces of both trapezoid and inverse trapezoid conditions showed no difference between the DOM and NDOM hands (see Supplementary Fig. S2a,b) in the supplementary material. A further investigation (see supplementary Fig. S2c,d) showed that the ΔV values were closer to 1, indicating a high covariation between the normal forces of the thumb and VF. Such a condition has been previously attributed to the task mechanics that arise from the equilibrium constraint (A perfect synergy situation due to the condition: Thumb normal force = Normal force of the virtual finger)^36^.

**Fig. 4:**
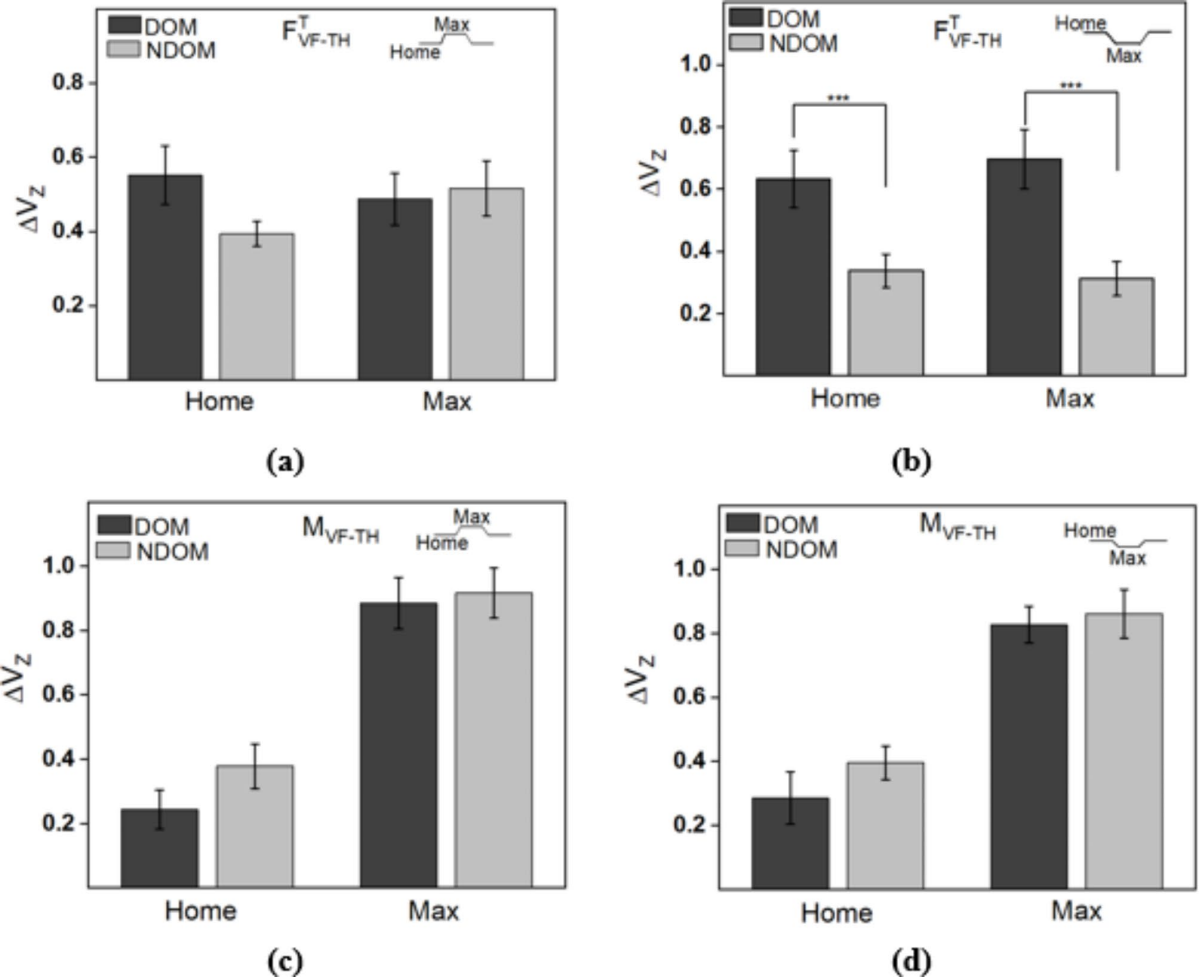
Average values of z-transformed synergy indices computed for (**a**) Tangential forces for trapezoid condition, (**b**) Tangential forces for inverse trapezoid condition, (**c**) Total moment for trapezoid condition, and (**d**) Total moment for inverse trapezoid condition. Error bars represent S.E.M. $\:{F}_{VF-TH}^{T}$ indicates that the synergy indices were computed using the tangential forces of the virtual finger (VF) and thumb (TH). Similarly, $\:{M}_{VF-TH}\:$indicates that the synergy indices were computed using the total moment obtained from the thumb and virtual finger forces.

In the case of tangential forces, no difference was observed in the computed synergy indices for the trapezoid condition (Fig. 4a) for either factor—position and hand. However, the inverse trapezoid condition showed a notable difference between the tangential force synergy index of the DOM and NDOM hand (Fig. 4b). A post hoc Tukey test showed that the synergy index of the tangential forces of the DOM hand was higher (*p* < 0.001) than the NDOM hand at both the MAX position (DOM: mean = 0.69, S.E.M = 0.09; NDOM: mean = 0.31, S.E.M = 0.055) as well as at the HOME position (DOM: mean = 0.63, S.E.M = 0.091; NDOM: mean = 0.33, S.E.M = 0.054). The ANOVA results showed a marked effect on the factor hand (F_(1,13)_ = 19.3, *p* < 0.01, *η*^2^ = 0.59) but no difference between the factor position and the interactions.

For both the trapezoid and inverse trapezoid conditions (Fig. 4c,d), there was no difference in the computed synergy indices of total moment between the DOM and NDOM hands. A two-way ANOVA performed on the synergy index of the total moment showed a considerable effect on the factor position (F_(3,11)_ = 191.46, *p* < 0.001, *η*^2^ = 0.93) and (F_(3,11)_ = 45.22, *p* < 0.01, *η*^2^ = 0.77) respectively. The ANOVA results showed no difference between the factor hand and the interactions. Surprisingly, the synergy values were lesser at the HOME position than the MAX position in both trapezoid and inverse trapezoid conditions.

**Fig. 5:**
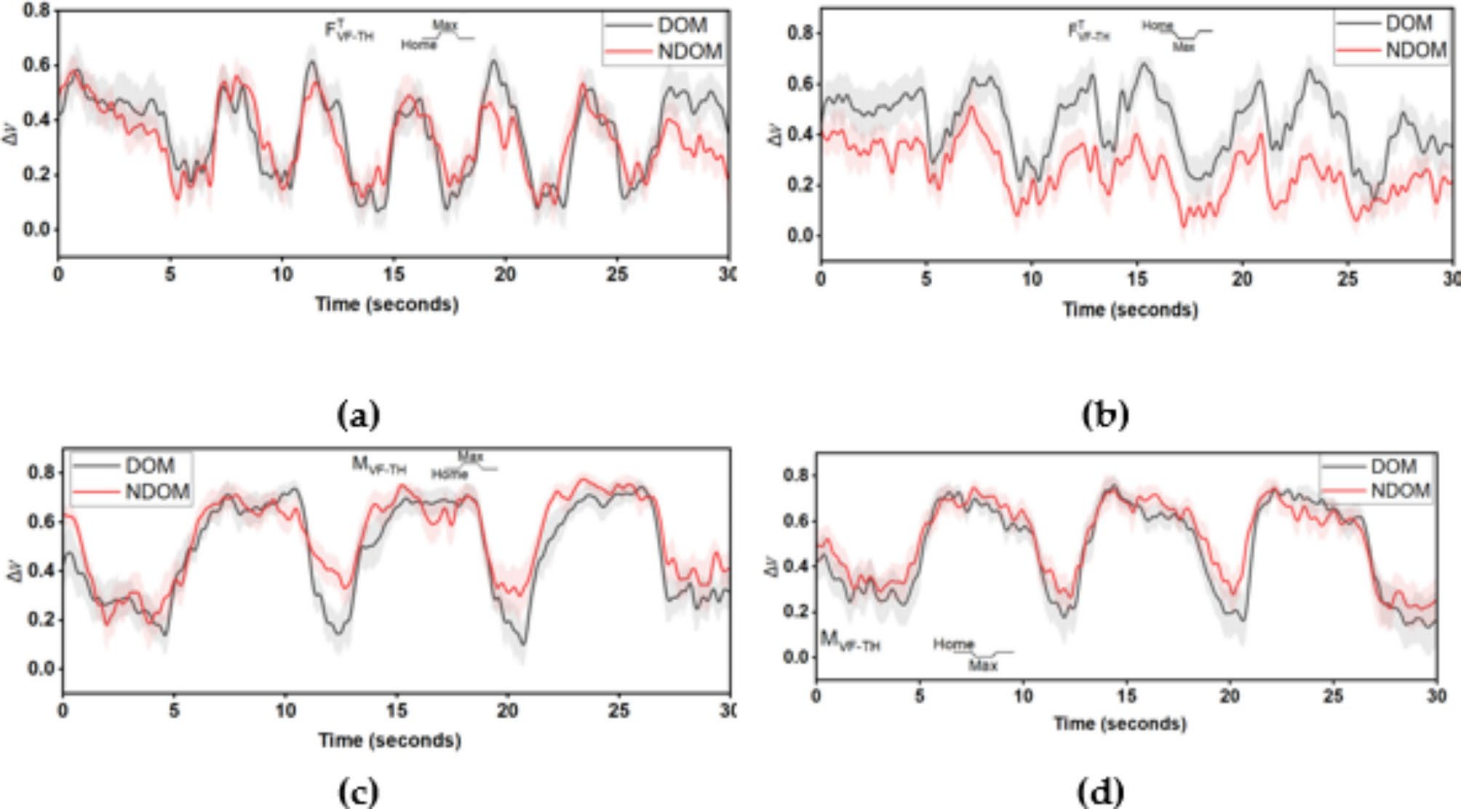
Comparison of tangential force synergy index between the DOM and NDOM hand averaged across all participants for (**a**) Trapezoid and (**b**) Inverse trapezoid condition. Comparison of total moment synergy index between the DOM and NDOM hand averaged across all participants for (**c**) Trapezoid and (**d**) Inverse trapezoid conditions. The shaded area represents the S.E.M.

Temporal Δ*V* values for the tangential forces and total moments of the trapezoid and inverse trapezoid condition were averaged across participants to visualize the variation in the synergy index of the tangential forces and total moments. The average synergy values with S.E.M for the tangential forces are depicted in Fig. 5a,b for trapezoid and inverse trapezoidal patterns, respectively. Similarly, the average synergy values with S.E.M for the total moment are depicted in Fig. 5c,d for trapezoid and inverse trapezoidal patterns, respectively. From the figure, it can be observed that for the inverse trapezoid condition, the tangential force synergy index of the NDOM hand remains lower than the DOM hand throughout the trial in all phases (up-ramp, down-ramp, and as well as in the static phase). As mentioned earlier, a drop in the total moment synergy index in the HOME position can be seen before the upramp/downramp movement begins.

### Analysis of anticipatory synergy adjustments

**Fig. 6:**
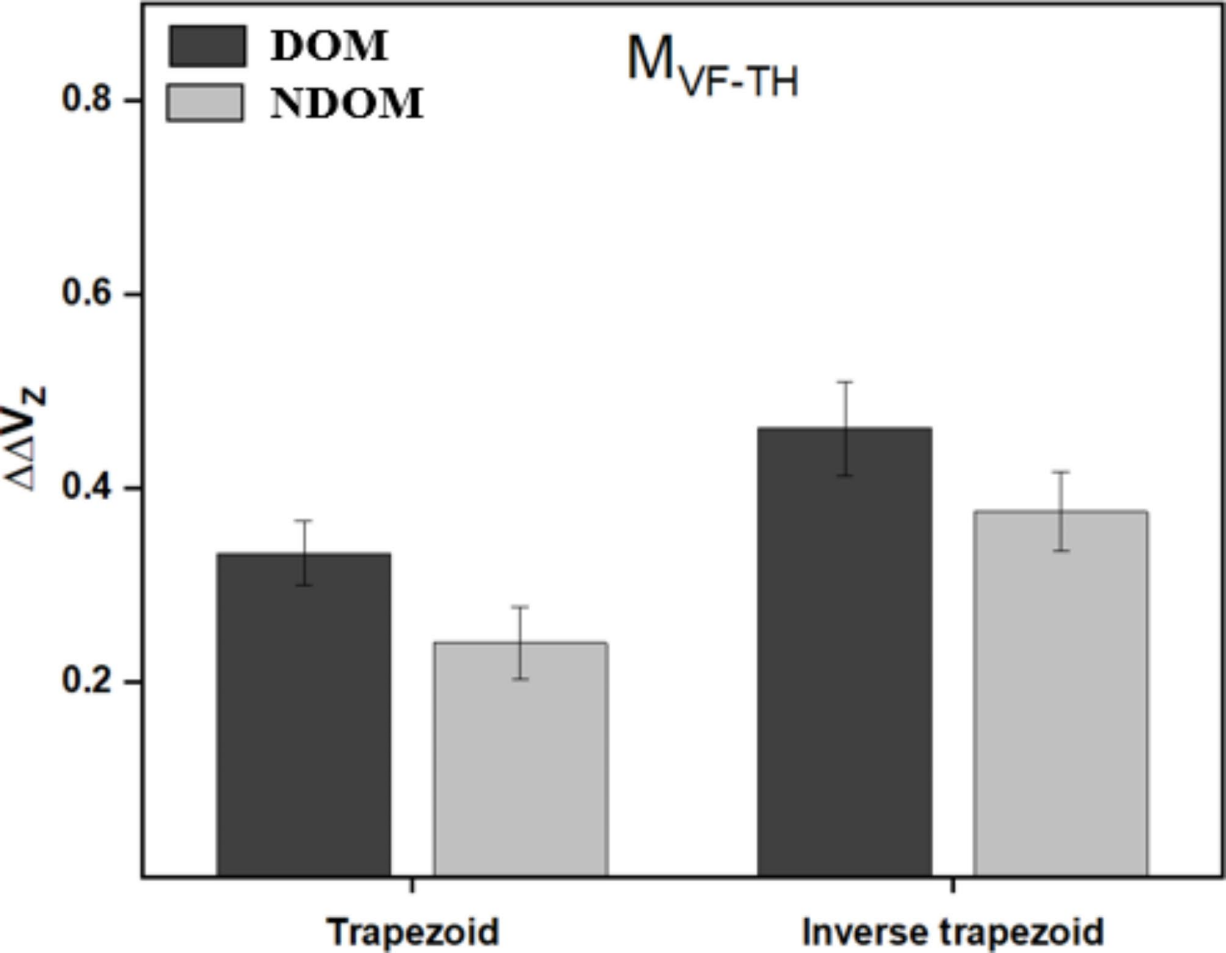
Comparison of$\:{\:\varDelta\:\varDelta\:V}_{z}$ values computed using the total moment synergy index for the DOM and NDOM hand. The analysis is performed for both trapezoid and inverse trapezoid conditions.

An analysis of ASA was performed to identify if the drop in the synergy index of the total moment at the home position (i.e., before the movement initiation towards the MAX position) was significantly greater for the DOM hand compared to the NDOM hand. The averaged values of $\:{\varDelta\:\varDelta\:V}_{z}$ computed for the synergy index of the total moment are shown in Fig. 6 for trapezoid and inverse trapezoid conditions. While the drop in synergy index was slightly higher for the DOM hand, statistically, no statistically notable difference was observed in the $\:{\varDelta\:\varDelta\:V}_{z}$ values between the DOM and NDOM hand.

## Discussion

This paper compared the prehensile synergies and force magnitudes of the DOM and NDOM hands during a 5-fingered prehension task. The participants performed a trapezoid and an inverse trapezoid position tracing task with their thumb while maintaining the handle in static equilibrium. Even though previous studies have shown mixed results regarding the performance of the DOM and NDOM hands in the finger space, as per the dynamic dominance hypothesis, the DOM hand was expected to perform better in stabilizing forces while performing movements than the NDOM hand. In the current study, the results matched with the dynamic dominance hypothesis only for the inverse trapezoid condition and not for the trapezoid condition. The synergy index for the NDOM was less than the DOM hand for the inverse trapezoid condition, indicating better coordination in the finger forces of the DOM hand. Additionally, even though the DOM and NDOM hands traced similar trajectories as per the task requirements, in the absence of visual feedback of the force, the little finger of the NDOM hand applied substantially greater grip force than the DOM hand only in the inverse trapezoid condition. Since the analysis was performed in the static region, it would be reasonable to expect the NDOM hand to perform better than the DOM hand. However, it should be observed that the position of the thumb achieves a pseudo-static position, continuously preparing for the next dynamic movements, resulting in continuous thumb displacements throughout the trial. This is visible in the displacement profile in Fig. 2.

Analysis of the tangential force synergies showed that the synergy indices were considerably greater for the DOM hand than the NDOM hand for the inverse trapezoid condition. Greater grip force and reduced synergy for the NDOM hand indicate diminished coordination between fingers to maintain the object’s rotational equilibrium compared to the DOM hand, which was in line with the dynamic dominance hypothesis. Why such a result was not observed for the trapezoid condition can probably be answered by the task demands.

Previously, our study that used a similar setup demonstrated that, for the DOM hand, the little finger exerted a greater amount of force when the thumb slider was moved from the HOME to the MAX position below the HOME (or towards the little finger—inverse trapezoid condition) when compared to the movement of the thumb from the HOME to the MAX position above the HOME position (or towards the index finger-trapezoid condition)^30^. The index finger was expected to produce a greater force than the little finger since the index finger is stronger than the little^37–39^. However, the little finger was observed to produce a greater force. The current study also demonstrated such a result. On similar lines, the differences between the hands were observed only in the inverse trapezoid condition and not in the trapezoid condition. In the same previous study, it was demonstrated that the downward movement of the thumb slider towards the little finger was associated with greater adduction of the little finger (which was apparent from the tangential forces). Such an effect was not observed in the index finger when the thumb moved upwards towards the index finger. Thus, the probable morphological reasons made the operation of the thumb in the vicinity of the little finger towards the extreme range of movement. This makes grasping and maintaining the object under equilibrium challenging when the thumb moves towards the little finger, resulting in increased task demands. Thus, on similar lines, the role of handedness was observed only in inverse trapezoid conditions while the fingers operated in an extreme range of movement, requiring greater force application. The DOM hand’s little finger required considerably less grip force to maintain the handle stable than the NDOM hand. The synergy index was also greater for the DOM hand than the NDOM hand. Thus, handedness-related differences for distal limbs are observed only in those cases with increased task demands.

Along with the framework for hemispherical control of the dominant and the non-dominant hand, the dynamic dominance hypothesis also proposed that each hand depends on both hemispheres to perform a movement, but with different contributions depending on the task demands^40^. As the task demands increase, specific roles of the hemispheres associated with handedness become more prominent. Many other studies have previously demonstrated the role of inter-hemispheric collaboration as task demands increase^41–43^. These studies have revealed that at lower task demands, there exists a collaboration between the hemispheres that results in the completion of the task. As task demand increases, the specific role played by the contralateral hemispheres becomes more prominent. For instance, by providing divided visual field stimuli at low complexity to either hemisphere, both hemispheres could complete the task independently^44,45^. However, at higher complexity, the role of the contralateral hemisphere dominated, thus showing a marked difference in the roles played by the hemispheres as task demands increased. Such a result has also been shown to be true using EEG measurements^46^. Another study demonstrated that prior knowledge of the responding hand is responsible for shifting the control from a default-focused dominant state to an interhemispheric configuration^47^. Thus, as familiarity with a particular task increases, the complexity is reduced, thus resulting in the interhemispheric mode of operation. Hence, in these lines, the differences were more prominent due to the probable complex nature of the inverse trapezoid task due to greater activation of the contralateral hemisphere than during the trapezoid task. This could also be why no difference in synergies was observed between the hands in previous studies performed in the kinematic space^26^ and the muscle synergy space^27^. These studies employed less demanding hand postures and grasps, which could have probably constrained the kinematics, thus not allowing any room for exploration of synergies.

Additionally, in the analysis of synergies of total moments, even though there were no statistically observable differences between the hands, some difference in the synergy index was observed for the MAX and HOME conditions. Surprisingly, the synergy values were lower in the HOME position than in the MAX position. Temporal synergy values were also plotted for visualization (Fig. 5c,d). The analysis revealed that there was a drop in synergy index before the beginning of each trapezoid, i.e., just before the thumb movements that follow an up ramp, max position, and down ramp (for trapezoid condition) and just before the thumb movements follow a down ramp, max position and up ramp (for trapezoid condition). In other words, the drop in synergy is observed only before the movement initiation and not during the actual movement. Such a phenomenon has been previously attributed to the anticipatory synergy adjustments^34,35^. A drop in the synergy index before the movement initiation indicates a greater destabilization of forces, mainly in response to a movement expectation. This drop in the synergy index is usually observed 150–400 ms before the actual move^34^. The greater the drop in synergy values during the movement preparation stage, the better the fingers’ ability to coordinate finger forces during the movement phase. In the current study, the temporal plot for the total moment showed an observable drop in the synergy index for the DOM hand than for the NDOM hand (Fig. 5c). An additional analysis was performed to investigate whether the destabilization was significantly greater for the DOM hand before the move. However, the results showed no significant difference in the drop in synergy index between the DOM and NDOM hands.

Finally, one limitation of the current study is that specific task demands could have also influenced the observed results compared to inherent handedness-related strategies. Our findings indicate that the inverse trapezoid condition, which involves operating the thumb at extreme ranges of motion, posed greater complexity and required more precise control. This may have highlighted differences between the dominant and non-dominant hands that were not as evident in the trapezoid condition. To address this, further studies are required involving a broader range of tasks involving both right and left-dominant participants that could help us understand how task complexity and handedness interact. Additional analysis involving neural network-based models^48^ could provide further insights into the control mechanisms for multi-finger coordination in tasks with increased complexity.

In conclusion, the DOM hand was observed to be better at coordinating forces than the NDOM hand in the inverse trapezoid condition. Greater task demands were attributed to the drop in the synergy index of the NDOM hand being greater than the DOM hand only for the inverse trapezoid condition and not in the trapezoid condition. The difference was insignificant even though synergies of the total moment of the DOM hand showed an observable drop compared to the NDOM hand due to anticipatory synergy adjustments. As an application, it should be tested if the synergies can be used to develop an objective metric for handedness instead of the standard Edinbourg’s handedness inventory that subjectively classifies handedness. Such an experiment should involve a large set of both right and left-dominant participants.

## Concluding comments

Differences in the control of the dominant and non-dominant limbs have been previously demonstrated for arm movements. Studies showing similar results for the finger movements and forces are limited with mixed results. This study employed a unique grasping handle with a frictionless thumb platform to apply perturbation by moving the thumb platform along trapezoidal and inverse trapezoidal paths. The performance of the DOM and NDOM hands in stabilizing the perturbation was studied by analyzing their synergies. The results showed no changes in synergy for the trapezoid condition, whereas differences in both force levels and synergies were observed in the inverse trapezoid condition. The requirement of the operation of the thumb in the regions of the extreme range of movements in the inverse trapezoidal condition was attributed to the increased task demands in performing the task, thus elucidating the difference between the dominant and non-dominant hands.

## Electronic supplementary material

Below is the link to the electronic supplementary material.

Supplementary Material 1

## Data Availability

The data collected for this study is available upon request by contact with the corresponding author.
