# Supplementary material for: Task demands modulate distal limb handedness: A comparative analysis of prehensile synergies of the dominant and non-dominant hand
Source: Sci Rep. 2024 Oct 26;14:25565. doi: 10.1038/s41598-024-75001-3 (PMC11514032; doi:10.1038/s41598-024-75001-3)
Supplement: Supplementary file 1 — Supplementary Material 1 [file 41598_2024_75001_MOESM1_ESM.docx]

**Supplementary material**

1. **Analysis of tangential forces**

Tangential forces for the DOM and NDOM hands are shown for the trapezoid condition (Figure S1(a)) and inverse trapezoid condition (Figure S1(b)). The results showed no difference between the DOM and NDOM hands for any fingers in both trapezoid and inverse trapezoid conditions. There was a marked effect on the factor fingers for the trapezoid task (F_(4,10)_=24.29, p<0.01, 𝜂2=0.6512) as well as for the inverse trapezoid task (F_(4,10)_=85.76, p<0.01, 𝜂2=0.868). ANOVA results on the factor conditions showed no difference in both cases. The interactions of factors fingers and position-hand conditions, however, showed a statistically notable effect for the trapezoid task (F_(4,10)_=33.08, p<0.01, 𝜂2=0.717) and inverse trapezoid task (F_(4,10)_=34.69, p<0.01, 𝜂2=0.727). However, a post hoc Tukey test revealed no difference between the DOM and NDOM hand tangential force in the trapezoid and inverse trapezoid tasks for MAX and HOME conditions.

| 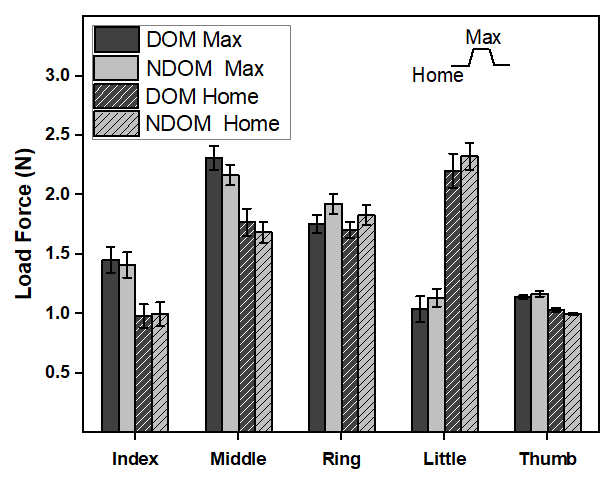 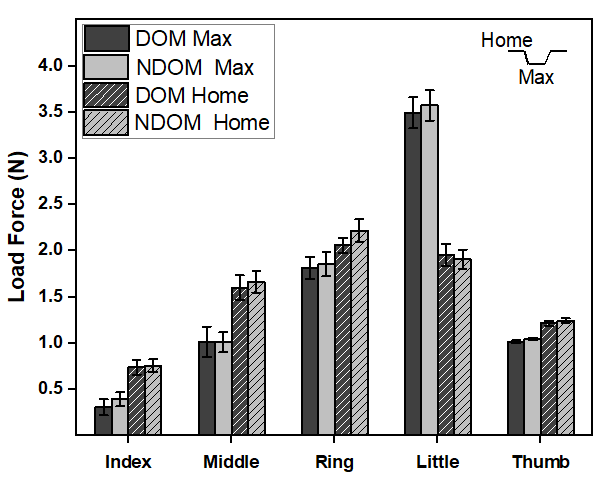 | |
| --- | --- |
| **(a)** | **(b)** |
| **Figure S1.** Average values of tangential forces for fingers and the thumb for (a) Trapezoid and (b) inverse trapezoid condition. Results are presented for the MAX and HOME positions for DOM and NDOM hands. Error bars represent S.E.M.   1. **Normal force synergies**   Synergy indices of the normal forces for both trapezoid and inverse trapezoid conditions are shown in Figure S2(a) and S2(b), respectively. ANOVA results showed no difference between the DOM and NDOM hand synergies for trapezoid and inverse trapezoid conditions. However, the results showed a considerable effect on the factor position (F_(1,13)_=18.33457, p<0.001, 𝜂^2^=0.5) for the trapezoid condition. Additional analysis was done to visualize Δ𝑉 values. These results are presented in Figure S2(c) and (d). The results show that the Δ𝑉 values are closer to 1, resulting in a perfect synergy condition.  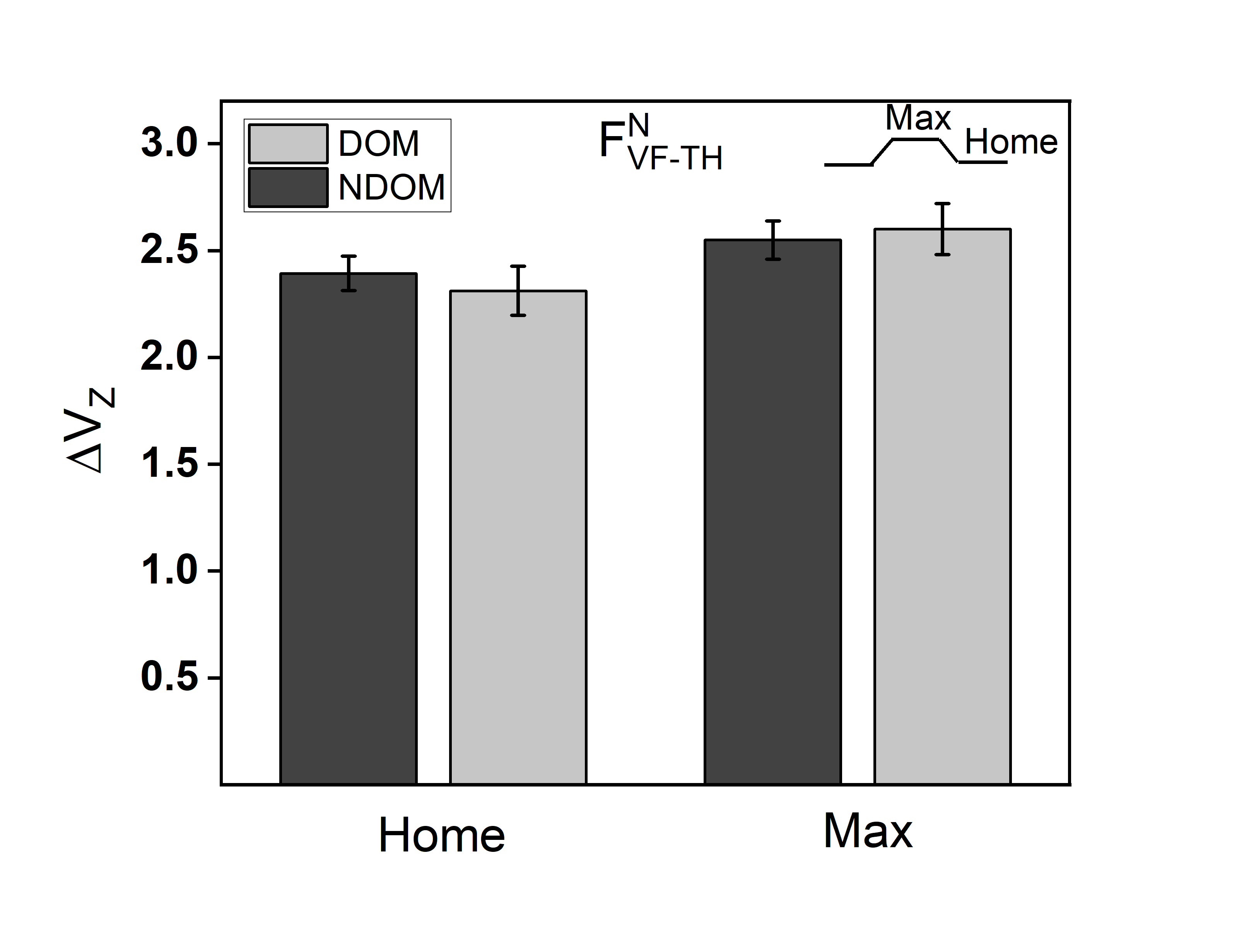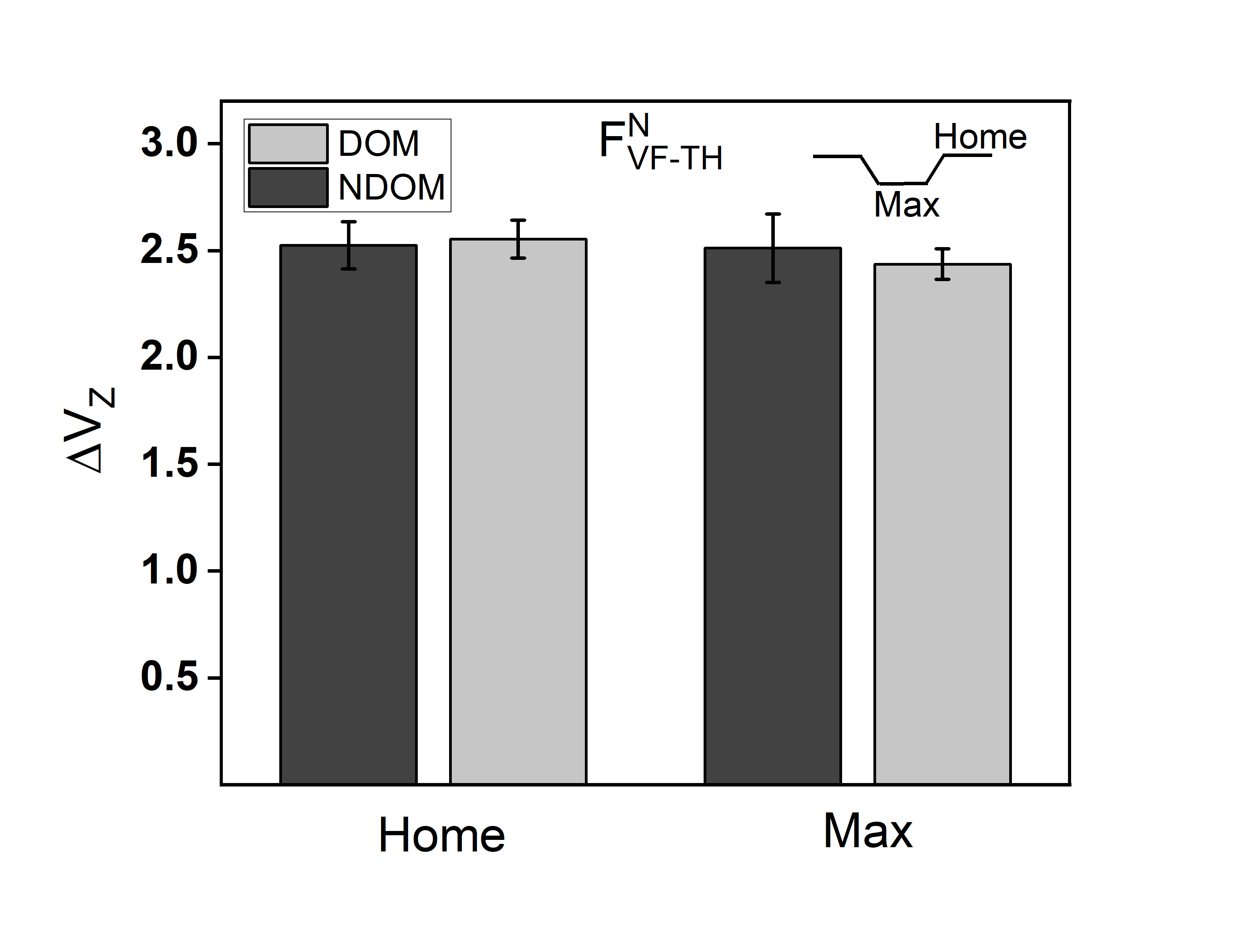   1. **(b)**   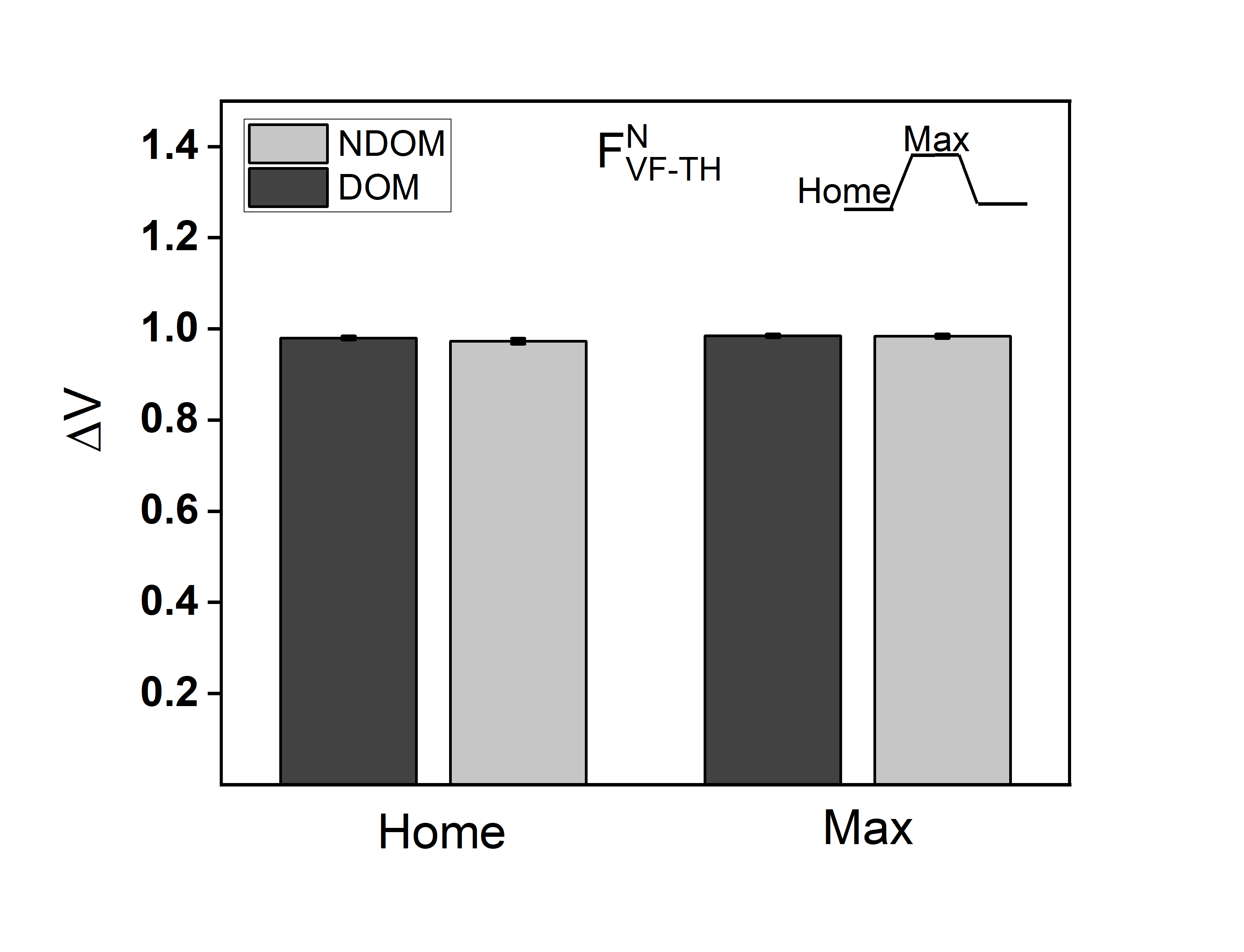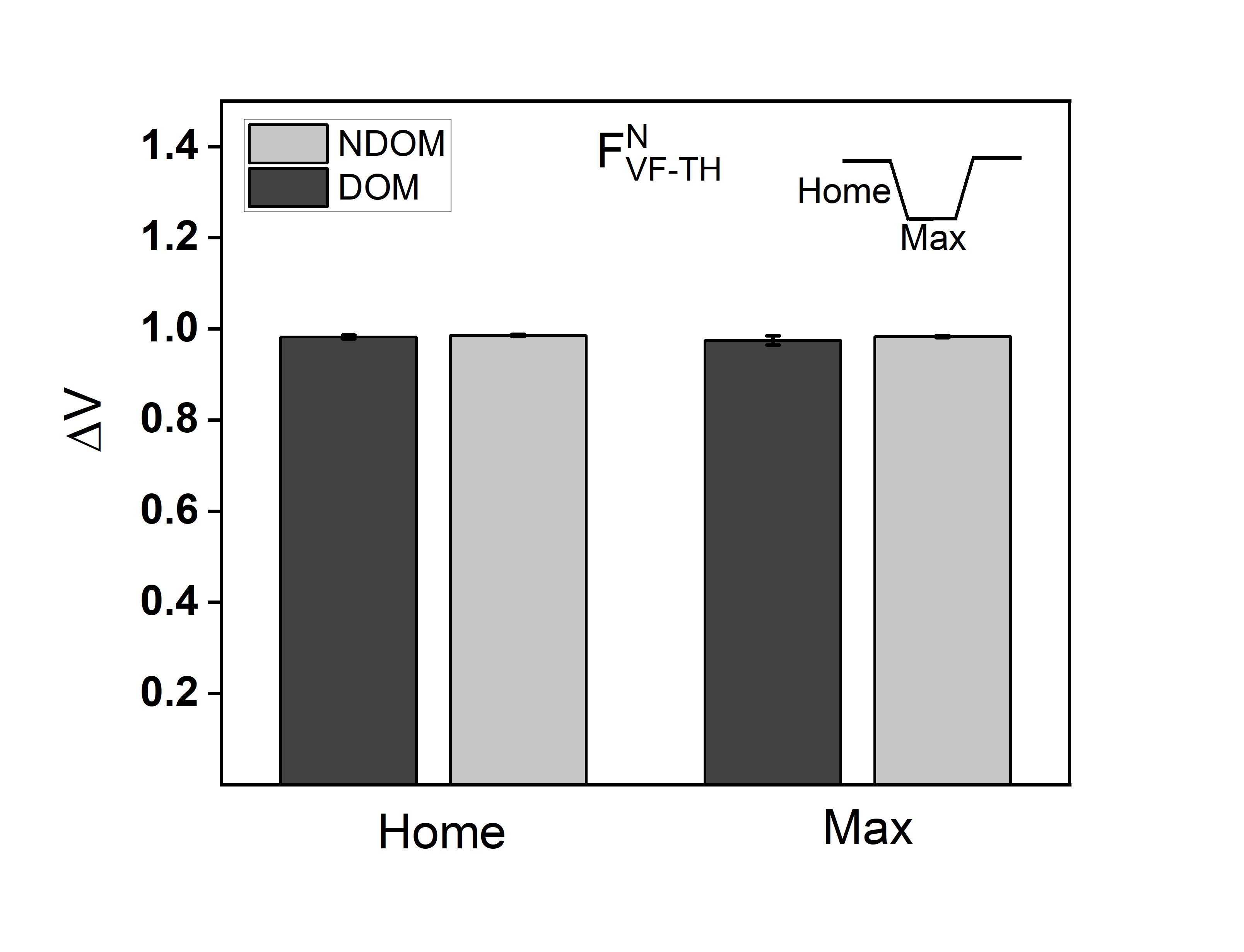  **(c) (d)**  **Figure S2** Average values of Z transformed synergies $\Delta Vz$ computed from the normal forces for (a) Trapezoid and (b) Inverse trapezoid condition. Average values of synergy index $\Delta V$ computed for (c) Trapezoid and (d) Inverse trapezoid condition. | |
